# A Cryptic Cytoplasmic Male Sterility Unveils a Possible Gynodioecious Past for *Arabidopsis thaliana*

**DOI:** 10.1371/journal.pone.0062450

**Published:** 2013-04-29

**Authors:** Nicolas Gobron, Cezary Waszczak, Matthieu Simon, Sophie Hiard, Stéphane Boivin, Delphine Charif, Aloïse Ducamp, Estelle Wenes, Françoise Budar

**Affiliations:** 1 INRA Institut National de la Recherche Agronomique, UMR1318, IJPB Institut Jean-Pierre Bourgin, Versailles, France; 2 AgroParisTech, IJPB Institut Jean-Pierre Bourgin, Versailles, France; Ecole Normale Superieure, France

## Abstract

Gynodioecy, the coexistence of hermaphrodites and females (i.e. male-sterile plants) in natural plant populations, most often results from polymorphism at genetic loci involved in a particular interaction between the nuclear and cytoplasmic genetic compartments (cytonuclear epistasis): cytoplasmic male sterility (CMS). Although CMS clearly contributes to the coevolution of involved nuclear loci and cytoplasmic genomes in gynodioecious species, the occurrence of CMS genetic factors in the absence of sexual polymorphism (cryptic CMS) is not easily detected and rarely taken in consideration. We found cryptic CMS in the model plant *Arabidopsis thaliana* after crossing distantly related accessions, Sha and Mr-0. Male sterility resulted from an interaction between the Sha cytoplasm and two Mr-0 genomic regions located on chromosome 1 and chromosome 3. Additional accessions with either nuclear sterility maintainers or sterilizing cytoplasms were identified from crosses with either Sha or Mr-0. By comparing two very closely related cytoplasms with different male-sterility inducing abilities, we identified a novel mitochondrial ORF, named *orf117Sha*, that is most likely the sterilizing factor of the Sha cytoplasm. The presence of *orf117Sha* was investigated in worldwide natural accessions. It was found mainly associated with a single chlorotype in accessions belonging to a clade predominantly originating from Central Asia. More than one-third of accessions from this clade carried *orf117Sha*, indicating that the sterilizing-inducing cytoplasm had spread in this lineage. We also report the coexistence of the sterilizing cytoplasm with a non-sterilizing cytoplasm at a small, local scale in a natural population; in addition a correlation between cytotype and nuclear haplotype was detected in this population. Our results suggest that this CMS system induced sexual polymorphism in *A. thaliana* populations, at the time when the species was mainly outcrossing.

## Introduction

Cytoplasmic male sterility (CMS) is known to spontaneously evolve in plant natural populations, where it leads to the coexistence of females (i.e. male-sterile individuals) and hermaphrodites. Such populations are called gynodioecious [1], [2]. In this reproductive system, the plant sexual phenotype results from the epistatic interaction between the cytoplasm, either sterilizing or not, and nuclear genes whose alleles may either restore male fertility in the presence of the sterilizing cytoplasm (restorer, Rf) or allow the expression of male sterility (maintainer). CMS is commonly thought to result from a genomic conflict between the maternally inherited cytoplasm and the Mendelian nuclear genome [3], [4].

The understanding of conditions behind the maintenance of sexual polymorphism in populations with CMS systems has motivated theoretical and population genetic studies for many years [4]–[8]. Some models propose pleiotropic effects of either sterilizing cytoplasms or nuclear restorers, or both, such as the cost of restoration and deleterious sterilizing cytoplasms [7], [8]. Fitness effects of CMS genetic factors may act even in the absence of sexual polymorphism. In addition, it has recently been argued that the pleiotropic effect of Rf alleles on fitness is modulated by interaction with ecological factors [9]. Although sexual polymorphism reflects genetic polymorphism at least in one genome compartment, the opposite is not true: there may be genetic polymorphism without any sexual polymorphism. For example, when restorer alleles are fixed in a species or population, the occurrence of the corresponding sterilizing cytoplasm is hardly discernible at the phenotype level: it has become cryptic [10]. Hermaphroditic species or populations that carry cryptic CMS can be unveiled only by crosses with unrelated genotypes not carrying the corresponding restorers. A case of cryptic CMS was recently revealed after hybridization of two Mimulus species whereas no gynodioecy was observed in *M. guttatus*, the sterilizing cytoplasm donor [11]. Nuclear restorers seem to be common in *M. guttatus*, although the sterilizing cytoplasm is almost exclusively restricted to the population where it was originally described [12]. Recently, it was suggested that CMS could be involved in male sterility of hybrids between European and American *Arabidopsis lyrata* populations [13], although to our knowledge no gynodioecy has ever been reported in this species.

It is therefore very likely that CMS has contributed to the coevolution of plant genetic compartments in more plant lineages than those in which gynodioecy is observed in natural populations. Our ability to address questions on how cytonuclear coevolution is shaped by cryptic CMS systems in hermaphroditic species obviously relies on our ability to trace cryptic CMS in populations, best achieved by knowing the causative genes. Although very few molecular factors have been identified in naturally occurring CMS [14], [15], studies on crops in which CMS is used to develop hybridization systems at the field scale have identified over a dozen sterilizing mitochondrial genes (for reviews, see [16]–[18]). Typically, CMS sterilizing inducers are unique mitochondrial genes and most of them result from genomic rearrangements. Nuclear restorers most commonly interfere with the expression of the mitochondrial sterilizing genes. A handful of nuclear restorers have been identified and most of them encode proteins belonging to the pentatricopeptide repeat (PPR) family. This protein family was first discovered after sequencing the *A. thaliana* genome [19], and has been shown to have expanded spectacularly in land plants [20]. PPR proteins act in multiple steps of organelle gene expression by virtue of their ability to interact with specific RNAs [21]. Recently, signals of diversifying selection were detected in genes encoding PPR proteins that are related to known Rfs [22]. They are taken as traces of the genomic conflict between mitochondrial and nuclear genomes in angiosperms. Interestingly, species-specific clusters of paralogous Rf-like PPR genes have been found in *A. thaliana* in which gynodioecy is very unlikely due to a predominantly selfing reproductive system [20], [22].

Nevertheless, we report here the occurrence of cryptic CMS in the hermaphroditic, selfing model plant *A. thaliana*. Taking advantage of the close similarity between a sterilizing and a non-sterilizing cytoplasm, we were able to identify a new mitochondrial gene most likely causing sterility. We analyzed the distribution of this gene in a global sampling of the species and in a natural population. The results show that the sterility-inducing cytoplasm is geographically restricted and that it can coexist with a non-sterilizing cytoplasm at a local scale. Both analyses revealed striking correlation between the sterilizing cytoplasm and nuclear haplotypes.

## Results

### Cytoplasmic Male Sterility Arose in a cross Between Distantly–related Arabidopsis Accessions

**Figure 1:**
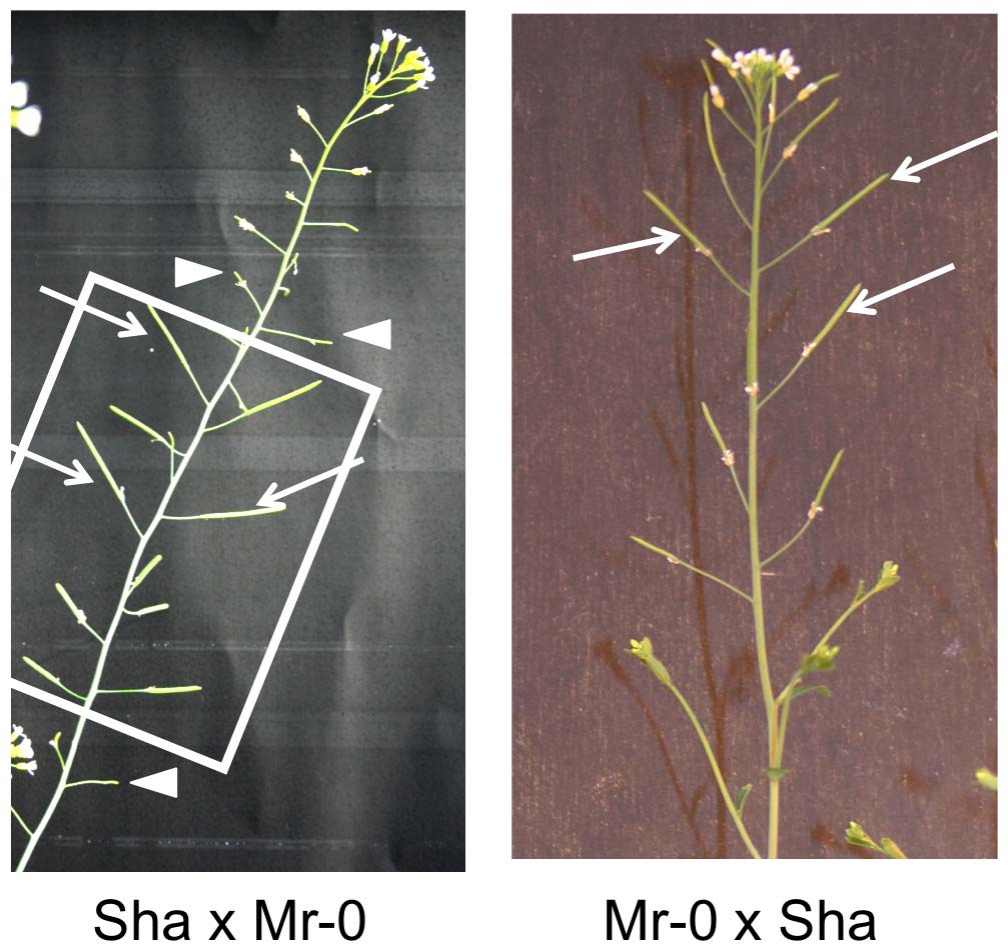
Phenotypes of reciprocal F1s from Sha and Mr-0 parents. Reciprocal F1s are shown at the flowering stage. The Mr-0 x Sha F1 (right panel) is fully fertile as shown by the elongated siliques containing developing seeds (arrows). The Sha x Mr-0 F1 (left panel) is sterile as shown by short remnants of pistils, producing no seeds (arrowheads). The boxed area highlights elongated siliques with developing seeds (arrows) resulting from hand-pollination of the flowers with pollen from a fertile plant.

In a series of systematic reciprocal crosses between distant accessions for the production of F2 families, Shahdara (Sha, originating from Tadjikistan) and Monte-Rosso (Mr-0, originating from Italy, see Plant Material) gave reciprocal F1s with different reproductive phenotypes: when Sha was used as the mother, the F1 plants were sterile, whereas the reciprocal F1s were fertile. The lack of seed set in sterile plants resulted from male sterility, as the seed production was restored when sterile flowers of the Sha x Mr-0 F1s were hand-pollinated with fertile plants (Figure 1).

Sterile Sha x Mr-0 plants were pollinated with both parents. Backcross with Mr-0 produced sterile progeny whereas backcross with Sha produced a segregating progeny, where about one-fourth (44 out of 189; Chi-square p = 0.59) of the plants were sterile (producing no seeds at all) or mainly sterile (producing very few seeds). We concluded that the association of the Sha cytoplasm with two dominant Mr-0 alleles was leading to pollen abortion, therefore revealing a case of cytonuclear male sterility (CMS), hereafter named Sha/Mr CMS.

**Figure 2:**
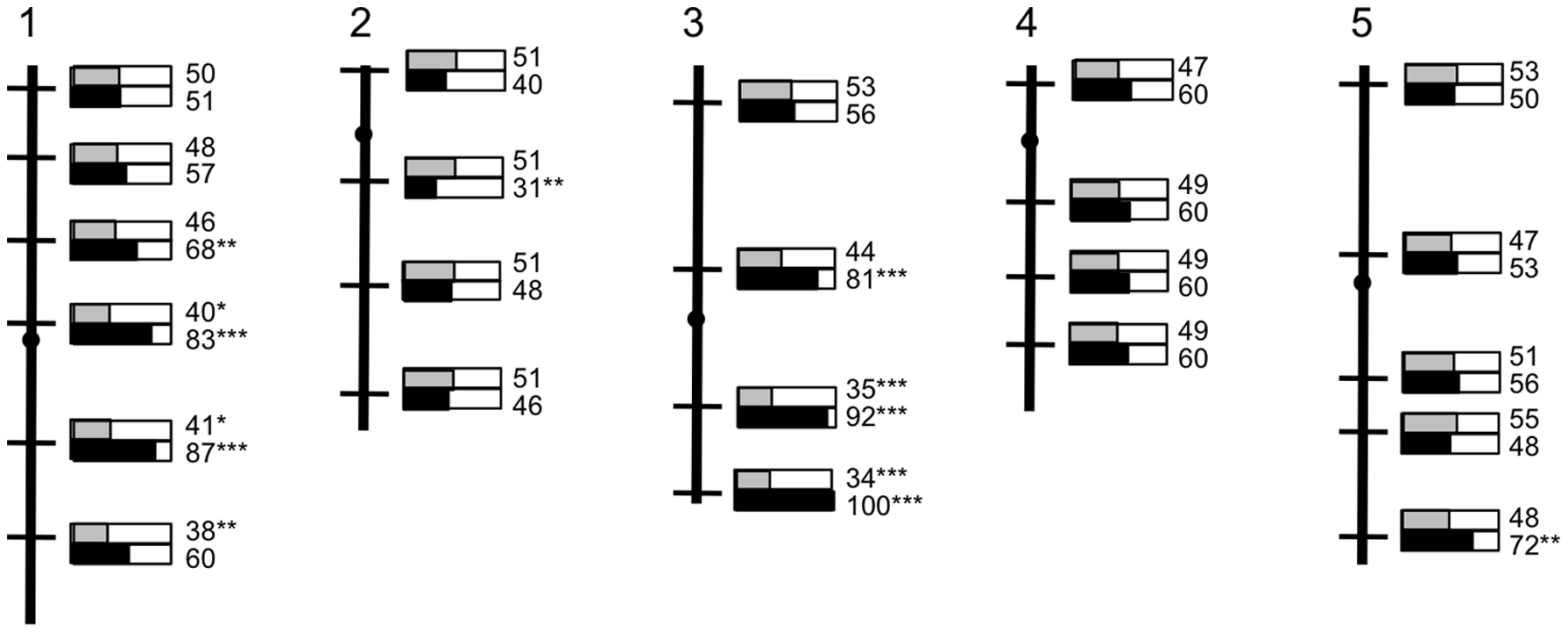
Genotyping of the (Sha x Mr-0) x Sha backcross population. The five Arabidopsis chromosomes are represented as vertical black bars, with centromeres indicated as dots. Markers used for genotyping are indicated as thin black cross-bars, according to their physical position on the chromosomes (see also Table S1). At each marker position, the gray and black sectors in the small bar graphs indicate proportions of heterozygotes found in the fertile (top) and sterile (bottom) plant subpopulations, respectively. These proportions are given as percentages on the right. Asterisks indicate proportions that significantly differ from the expected 50% of heterozygotes (* 0.05>P>0.01; **0.01>P>0.001; *** P<0.001).

As the Sha x Mr-0 F1 was sterile, we inferred that, at loci controlling the sterility phenotype, Mr-0 maintainer alleles were dominant on Sha restorer alleles. The segregating backcross population was genotyped for 23 microsatellite markers spread over the genome. When the population was considered globally, the proportion of genotypes was not significantly different from the expected 1∶1 ratio for each marker. We reasoned that loci controlling the sterility phenotype should be heterozygous in sterile progeny and fixed for Sha alleles in the fertile progeny. When the population was divided into subpopulations of fertile or sterile individuals, markers on chromosomes 1, 3 and –to a lesser extent– 5 appeared biased, with an excess of heterozygotes among sterile plants, and markers on chromosome 1 and 3 presented an excess of Sha homozygous alleles among fertile ones (Figure 2, Table S1). Since we expected only two causative loci to be involved according to the segregation of the sterility phenotype, we closely examined the genotypes of the plants and found linkage disequilibrium between the marker on the southern end of chromosome 5 and markers on the southern end of chromosome 3. Because the latter were strongly correlated with the phenotype, it is very likely that the mild bias observed at chromosome 5 resulted from the linkage disequilibrium between these two regions. We therefore hypothesized that chromosomes 1 and 3 carry the Mr-0 alleles necessary for the sterility phenotype.

**Figure 3:**
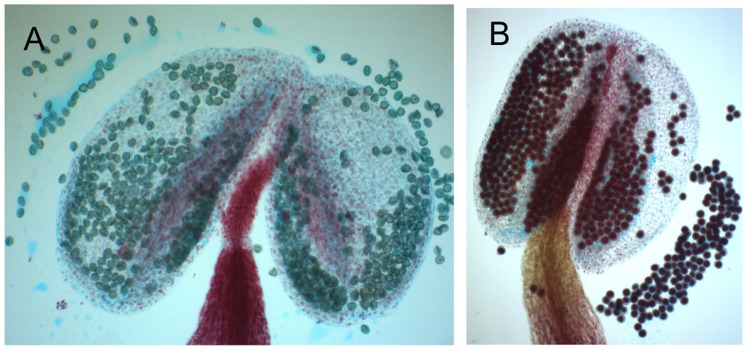
Alexander staining of anthers on plants from the fourth backcross. Anthers were dissected from buds just before opening, stained with Alexander’s staining, mounted on glass slides and observed under a light microscope. A: typical anthers from a sterile plant. B: typical anther from a fertile plant. Red-colored pollen is viable, whereas blue-green pollen has aborted.

We introgressed the two suspected regions into a Sha genetic background by performing three additional backcrosses with Sha, selecting a sterile plant as the mother of the cross at each generation. Heterozygosity at markers linked to maintainer genes on chromosomes 1 and 3 was verified at each generation. Two dozen plants of the fourth backcross population were grown, and again about one-quarter of them were sterile. Sterile plants were heterozygous at markers on chromosomes 1 (MSAT1.21231 and ATHGENEA) and 3 (MSAT3.23007). They were fixed for Sha alleles at markers on chromosome 5 (MSAT5.20037 and MSAT5.19). Anthers of fertile plants contained viable pollen (dark red after Alexander staining), whereas anthers of sterile plants were full of aborted pollen (blue-green stained) (Figure 3). We concluded that the southern arms of chromosome 1 and 3 carried the nuclear genes involved in the Sha/Mr CMS.

### Maintainer Alleles are Shared by Several Accessions

The smallest core-collection of Versailles Arabidopsis Stock Centre [23] includes Sha, and seven additional accessions, namely Blh-1, Bur-0, Ct-1, Cvi-0, Ita-0, Jea, and Oy-0. These accessions were used in reciprocal crosses with Sha, and all reciprocal F1s obtained were fully fertile. After three backcrosses with the father of the first cross, only the Sha/Cvi-0 combination was male-sterile, indicating that Cvi-0 contained recessive maintainers, whereas all the others contained restorers of fertility.

We also searched for other accessions with maintainer alleles among those genetically close to Mr-0. Etna-1 and Etna-2, both originating from Sicily, were the accessions most closely-related to Mr-0, based on the SNP polymorphisms used to characterize the genetic resources available in Versailles [24].

Reciprocal F1s obtained from Sha and Etna-2 gave similar phenotypes to those from Sha and Mr-0 (male sterile Sha x Etna-2 F1 and fertile Etna-2 x Sha F1; Figure S1). The backcross of Sha x Etna-2 by Sha gave approximately one-quarter (43 out of 141) of sterile or mainly sterile plants. The genotyping of this segregating population on the five chromosomes revealed an excess of heterozygotes in the sterile subpopulation for the three markers tested on chromosome 1 (CIW12, NGA128, and ATHGENEA), for two markers on chromosome 3 (MSAT3.1 and MSAT3.65), and for one marker on the southern arm of chromosome 5 (ICE3). In the fertile subpopulation, we found an excess of heterozygotes on the northern end of chromosome 3 (MSAT3.02502) and an excess of Sha homozygote alleles on the northern end of chromosome 5 (NGA158). This latter marker was also biased in the whole backcross population, which showed a global excess of plants homozygous for the Sha allele at this position (Table S2; Figure S2).

Both Sha x Etna-1 and Etna-1 x Sha F1s died a few days after germination (Figure S3). As this phenotype was identical in both F1 progenies, we suspect that it was not linked to cytonuclear interactions, but rather to allelic incompatibilities similar to those previously described in *A. thaliana* [25], [26]. However, the premature death of F1 plants prevented direct testing of the presence of maintainer alleles in Etna-1. We therefore used a Mr-0 x Etna-1 F1 as a pollen donor in a cross with Sha. Half (99/191) of the progeny died after germination, indicating that a single locus in Etna-1 controlled the lethality of Sha/Etna-1 hybrids. None of the surviving plants was fully fertile, most were sterile (61/82) and the others were mainly sterile (Figure S4). The surviving plants carried Mr-0 and Etna-1 alleles in balanced proportions at SNP markers tightly linked to Mr-0 maintainer loci, indicating that plants carrying the Sha/Etna-1 genotype at these loci were sterile (Table S3).

We concluded that maintainer alleles for the Sha/Mr CMS were also present in Etna-1 and Etna-2 accessions.

### Several Accessions Possess a Sterilizing Cytoplasm

At the same time, we tested cytoplasms from other accessions for their ability to induce male sterility in combination with Mr-0 nuclear alleles. Twenty accessions, geographically linked to Sha, or whose cytoplasms were previously found close to that of Sha [27], were crossed with Mr-0 both ways. Ten produced a sterile F1 when used as female in the cross (Table 1). All the accessions with a sterilizing cytoplasm possessed the same chlorotype, named ‘I’ according to our previously reported study on cytoplasmic diversity [27]. Conversely, among the 10 accessions whose cytoplasm did not induce sterility, only Kz-9 had the I chlorotype (Table 1). Previous analyses showed that the joint loss of three non-conserved adjacent mitochondrial ORFs, taken as a single structural variation, is typical of the I chlorotype [27]. We tested for this mitochondrial rearrangement in I chlorotype accessions used in crosses with Mr-0 by PCR-amplifying two of the ORFs, *orf240a* and *orf107d*. None of the sterility-inducing accessions contained any of these ORFs, whereas *orf240a* was detected in Kz-9 (Table 2). This result pointed to a difference between the mitochondrial genome of Kz-9 and those of accessions with sterilizing cytoplasms. All accessions with the I chlorotype, including Kz-9, were identical for the other previously described mitochondrial polymorphisms [27].

**Table 1:** Accessions tested in reciprocal crosses with Mr-0.

			Phenotype of F1s^a^			
Accession name	Versailles Identification number	Country oforigin	Acc x Mr-0	Mr-0 x Acc	Chlorotype^b^	Reference for chlorotype
9481B	261AV	Kazakhstan	**St**	Fe	I	[27]
Dja-1	534AV	Kyrgyzstan	**St**	Fe	I	This work
Dja-5	535AV	Kyrgyzstan	**St**	Fe	I	This work
Kar-1	531AV	Kyrgyzstan	**St**	Fe	I	This work
Kar-2	532AV	Kyrgyzstan	**St**	Fe	I	This work
Kyr-1	538AV	Kyrgyzstan	**St**	Fe	I	This work
Shahdara	236AV	Tadjikistan	**St**	Fe	I	[27]
Neo-3	539AV	Tadjikistan	**St**	Fe	I	This work
Sus-1	533AV	Kyrgyzstan	**St**	Fe	I	This work
Zal-1	536AV	Kyrgyzstan	**St**	Fe	I	This work
Zal-3	537AV	Kyrgyzstan	**St**	Fe	I	This work
Kz-9	403AV	Kazakhstan	Fe	Fe	I	This work
Db-1	132AV	Germany	Fe	Fe	J	[27]
Kz-1	402AV	Kazakhstan	Fe	Fe	J	This work
N13	266AV	Russia	Fe	Fe	J	[27]
Wil-1	72AV	Lithuania	Fe	Fe	J	[27]
Hodja-Obi-Garm	203AV	Tadjikistan	Fe	Fe	S	[27]
Hodja-Obi-Garm bis	270AV	Tadjikistan	Fe	Fe	S	This work
Kondara	190AV	Tadjikistan	Fe	Fe	S	[27]
Neo-6	540AV	Tadjikistan	Fe	Fe	U	This work
Sorbo	238AV	Tadjikistan	Fe	Fe	F	[27]

a The left column indicates the phenotype of the F1 when Mr-0 was the male parent, and the right column indicates the phenotype of the F1 when Mr-0 was the female parent.

St = male sterile; Fe = fertile.

b Chlorotypes are designated according to the letter code defined in Moison et al. [27].

**Table 2:** Typing of the Sha-specific mitochondrial rearrangement in cytoplasms of the I chlorotype.

Accession name	Versailles Identification number	*orf240a*	*orf107d*	Reference
Shahdara	236AV	absent	absent	[27]
9481B	261AV	absent	absent	[27]
Kar-2	532AV	absent	absent	This work
Sus-1	533AV	absent	absent	This work
Dja-1	534AV	absent	absent	This work
Dja-5	535AV	absent	absent	This work
Zal-1	536AV	absent	absent	This work
Kyr-1	538AV	absent	absent	This work
Neo-3	539AV	absent	absent	This work
Kz-9	403AV	**present**	absent	This work

### A New Mitochondrial Gene Present only in Sterilizing Cytoplasms

The above results suggested that the cytoplasm of Kz-9, although non-sterilizing, is closely related to that of Sha. We explored high-throughput sequencing data available for Sha [28] (www.1001genomes.org) for predicted polymorphisms in chloroplast and mitochondrial genes (mainly in exons), and checked them by sequencing the corresponding regions in Sha and Kz-9. We sequenced 11 chloroplast fragments and 24 mitochondrial fragments (∼24 kb in total). Among 69 verified polymorphisms (16 in the chloroplast and 53 in mitochondria), only three positions (2 in the chloroplast and 1 in the mitochondria) differed between Sha and Kz-9. The mitochondrial variation destroyed an *Eco*RI site in the *rpl5-cob* intergenic region of Sha (see below). Kz-9′s *ycf1* chloroplast gene possessed a non-synonymous variation, whereas that found in Sha’s *rpoC2* chloroplast gene was silent. Therefore, none of the verified SNPs was likely to be the cause of the Sha/Mr male sterility. Table S4 summarizes polymorphisms found in Sha and Kz-9 relative to the reference sequences for *A. thaliana* chloroplast and mitochondrial genomes, and their predicted effect on the encoded proteins.

**Figure 4:**
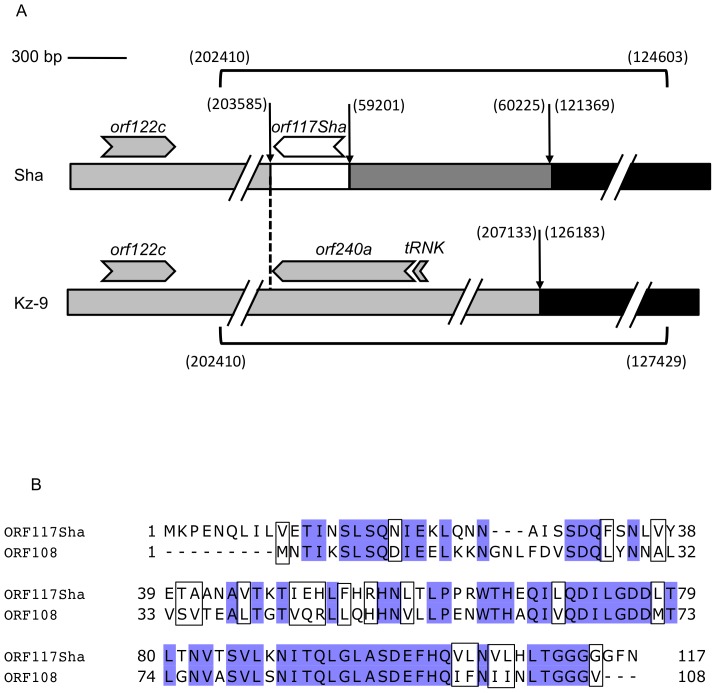
Variable mitochondrial genomic region in Sha and Kz-9. A. Schematic representation of the mitochondrial genomic regions that are organized differently in Sha and Kz-9 as revealed by DNA hybridization and sequencing. The white block indicates the 400 bp of unknown origin. The light gray blocks correspond to the *orf122c* region. The dark gray block is homologous to the *rpl5-cob* intergenic region. The black blocks correspond to the upstream region of *atp8*. Discontinuities in the scheme indicate colinear sequences not represented for scale convenience. Vertical arrows indicate breaks of colinearity with the reference sequence (Accession NC_001284); reference positions of the breakpoints are given in parentheses. The vertical dotted line indicates the point of divergence between the two sequences. Open reading frames are represented by arrows indicating the direction of translation. Horizontal brackets show parts that were sequenced (EMBL accessions HF543671 & HF543672), the corresponding positions of endpoints in the reference sequence are given in parentheses. B. Peptide alignment between ORF117SHA and ORF108. One-letter-code sequences of ORF117SHA predicted peptide and ORF108 [31] were aligned with Clustal 2.0.3 at phylogeny.fr [56] and the alignment was edited with Jalview [57]. Identical residues are shaded, residues with similar physicochemical properties are boxed [58].

Our initial analysis on cytoplasmic markers pointed to the loss of *orf240a* as a difference between sterility-inducers and Kz-9, suggesting a specific organization of sterility-associated mitochondrial genomes at this position. Plant mitochondrial genome diversity, at the species and within-species levels, mainly results from sequence rearrangements [29], [30]. In addition, most described CMS genes are non-conserved ORFs thought to result from such mitochondrial structural variations [18]. We therefore explored mitochondrial DNA structural variations between Sha and Kz-9 by comparing DNA hybridization profiles of both accessions with 23 gene probes (listed in Table 3). We calculated that our hybridization experiments scanned approximately 280 kb, which represented over three-fourths of the size of the *A. thaliana* mitochondrial genome of reference. Only four probes detected RFLP polymorphisms between Sha and Kz-9 (*trnK*, *orf122c, cob*, and *atp8*, Table 3 and Figures S5, S6, S7, S8). The *cob* probe displayed polymorphic hybridizing fragments only on *Eco*RI digests (Figure S6A), and this was further shown to result from a single nucleotide polymorphism that destroys an *Eco*RI site in Sha (see above, Table S4). *orf122c* and one copy of *trnK* are in the vicinity of *orf240a*, hence these two probes detected the same genomic rearrangement, which resulted in the loss of one *trnK* copy and *orf240a* in Sha (Figure 4A). With the *atp8* probe, Sha displayed the expected profile, whereas that of Kz-9 was different.

**Table 3:** Comparisons of mtDNA hybridization profiles in Sha and Kz-9 with mt gene probes.

Target gene	5′-3′ positions of probe^a^	Comparison between Sha and Kz-9profiles	Correspondingfigure
*orf107a*	36760–36952	identical	Figure S8B
*cox2*	42108–42353	identical	Figure S8A
*rpl5*	57850–58250	identical	Figure S7A
***cob***	**60471–60735**	**RFLP detected only with ** ***Eco*** **RI** b	**Figure S6A**
*nad6*	76665–77242	identical	Figure S8A
*atp6-1*	111930–112340	identical	Figure S7A
***atp8***	**130003–130231**	**RFLPs detected with ** ***Nco*** **I, ** ***Pvu*** **II and ** ***Spe*** **I** c	**Figure S7A**
*nad7*	135958–136207	identical	Figure S7A
*rps4*	159082–159620	identical	Figure S8B
*nad4*	161698–162048	identical	Figure S8A
***orf122c***	**201782–202057**	**RFLPs detected with ** ***Bam*** **HI, ** ***Eco*** **RI,** ***Hind*** **III, ** ***Pvu*** **II and ** ***Xba*** **I** d	**Figure S5B**
***trnK***	**204001–204831** e	**hybridizing bands corresponding to** **the copy in the vicinity of ** ***orf240a*** **are missing in Sha** f	**Figure S5A**
*cox3*	218309–218976	identical	Figure S7B
*rpl2*	220795–220974	identical	Figure S8B
*rpl16*	25080–25564	identical	Figure S8A
*ccb203*	257078–257299	identical	Figure S7B
*nad3*	260745–261019	identical	Figure S7A
*atp9*	278935–279107	identical	Figure S7B
*atp1*	302175–302838	identical	Figure S8A
*ccb256 ( = ccmC)*	307344–307682	identical	Figure S8B
*rps7*	314757–314999	identical	Figure S7B
*cox1*	350249–350527	identical	Figure S7A
*nad4L*	360596–360858	identical	Figure S8B
***orf117Sha***	**1173**–**1564**	**present only in Sha**	**Figure S6B**

a The 5′ and 3′ end positions of the probes are given according to the sequence of reference for the *A. thaliana* mt genome (NC_001284), except for *orf117Sha*. Position of the *orf117Sha* probe is given according to the sequence HF543671.

b This was further shown to result from a SNP at position 60081, which destroys an *Eco*RI site in Sha (Table S4).

c Sha has the expected profile, Kz-9 the variant profile.

d Kz-9 has the expected profile except with *Eco*RI and *Pvu*II, Sha the variant profile.

e The probe also hybridizes the other *trnK* copy at position 28709–29108.

f The common hybridizing fragments, corresponding to the *trnK* copy at position [28709–29108], are of the expected sizes; the Kz-9 bands corresponding to the *trnK* copy in the vicinity of *orf240a* are of expected size for *Hin*dIII, *Spe*I and *Xba*I digestions, not for *Bam*HI, *Eco*RI, *Pst*I and *Pvu*II.

The sequence of the rearranged regions in Sha and Kz-9 (EMBL accessions HF543671 & HF543672, respectively) showed that the regions downstream of *orf122c* and upstream of *atp8* were in close vicinity in both accessions. Therefore, all the mitochondrial structural variations detected in DNA hybridization experiments pointed to the same single region (Figure 4A). The Sha sequence showed the additional presence of 1025 bp from the *cob-rpl5* intergenic region (59201–60225) and contained 400bp of unknown origin (no hits in the public nucleotide databases after a Blast search). Interestingly, this unknown fragment contained an ORF potentially encoding a peptide of 117 amino acids, that we named *orf117Sha*. The predicted ORF117SHA peptide was found 56% identical and 69% similar to the ORF108 peptide of the *Moricandia arvensis* mitochondria, suspected to induce male sterility in *Brassica juncea* [31] (Figure 4B). DNA hybridization showed that *orf117Sha* is absent from the Kz-9 cytoplasm (Figure S6B). The presence of *orf117Sha* was further tested by PCR in the accessions crossed with Mr-0 (Table 1). All and only accessions whose cytoplasm induced male sterility gave positive amplification of *orf117Sha*. We concluded that *orf117Sha* was certainly the sterilizing factor of the Sha/Mr CMS.

### *orf117Sha* is Restricted to a Group of Related Accessions from Central Asia and Russia

To get a view of its distribution within the species, we checked the presence of *orf117Sha* by PCR amplification in 219 additional accessions (Table S5). Twenty-one of these accessions possessed *orf117Sha*.

Among the newly tested accessions, 87 had been previously analyzed for chloroplast markers and their chlorotypes were therefore already known [27]. We determined the chlorotypes of most remaining accessions and observed that *orf117Sha* was mainly associated with the I chlorotype (19 additional cases), and less frequently with the closely related J chlorotype (2 cases) (Table S5).

**Figure 5:**
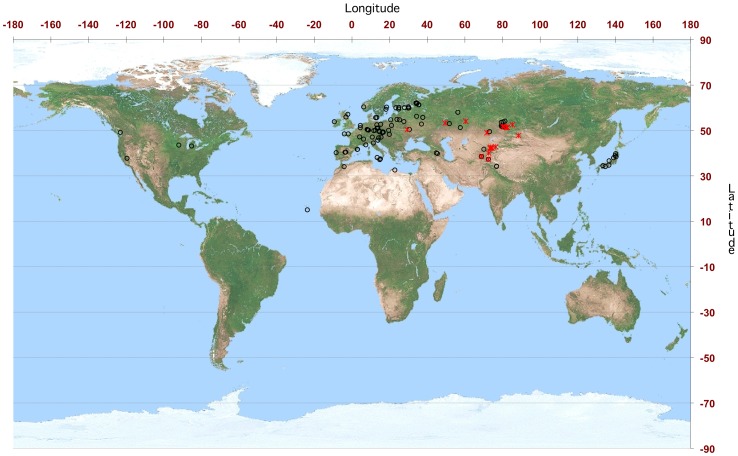
Geographical distribution of accessions tested for the presence of *orf117Sha.* Accessions are mapped to their geographical origin (information available on the Versailles Arabidopsis Stock Centre website http://dbsgap.versailles.inra.fr/vnat/). Accessions with *orf117Sha* are represented with red crosses, other accessions with black open circles.

**Figure 6:**
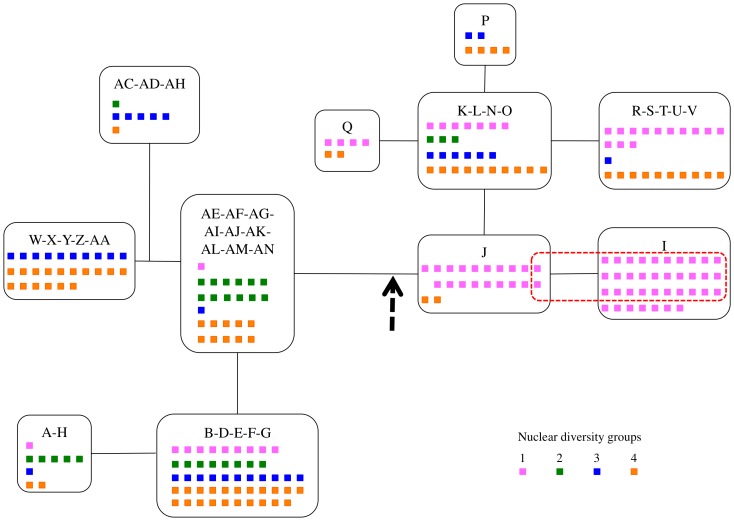
Distribution of nuclear diversity groups and of *orf117Sha* in a simplified chloroplast phylogeny. Chlorotypes or groups of chlorotypes are organized according to their phylogeny [27]. The vertical thick dashed arrow indicates the root of the network (according to [27]). Each small square represents an accession, colored according to its nuclear diversity group (pink: group 1, green: group 2, blue: group 3, orange: group 4) [24]. The red dotted line gathers accessions carrying the *orf117Sha* gene (see also Table S5).

Accessions used in this study fall into four distinct groups of nuclear diversity [24] (see also Materials & Methods). Interestingly, all the accessions carrying the *orf117Sha* belonged to group 1, which contained primarily accessions from Central Asia and Russia [24] (Table S5). The geographical distribution of accessions possessing *orf117Sha* showed that they originated mainly from Central Asia (Figure 5). Up to 35% (32 out of 91 tested) of accessions from the nuclear diversity group 1 possessed *orf117Sha*, whereas none of the accessions from the other groups did (Table 4). Figure 6 shows the distribution of nuclear diversity groups and that of the mitochondrial *orf117Sha* in a simplified chloroplast phylogeny.

**Table 4:** Distribution of accessions carrying *orf117Sha* according to nuclear genetic structure.

Nuclear genetic group^a^	Number of accessions tested	positive
1	91	32
2	31	0
3	42	0
4	78	0

a According to [24].

### Distribution of the Sterilizing Cytoplasm in a Natural Population

During our search for accessions carrying a sterility-inducing cytoplasm, we observed two accessions derived from the same neo-Sha population (see details in Plant Material) that gave different results: neo-3 possessed a sterility-inducing cytoplasm and an I chlorotype, whereas neo-6 carried a U chlorotype and its cytoplasm did not induce sterility (Table 1). Interestingly, these two accessions were also distinguishable by morphological differences and nuclear polymorphisms, although very closely related [24], [32]. We tested 23 additional individuals sampled from the same neo-Sha population for the presence of the *orf117Sha*, *orf240a* and *orf107d* mitochondrial ORFs, and determined their chlorotypes. Ten possessed the neo-3 signature for all tested polymorphisms, whereas the 13 remaining ones had the neo-6 profile. In addition, we found highly congruent structures for nuclear and cytoplasmic genetic diversities in the neo-Sha population: all individuals with the U chlorotype had a neo-6-like nuclear genotype whereas individuals with the I chlorotype and the *orf117Sha* mitochondrial ORF had a neo-3-like nuclear genotype, except neo-23 which had alleles from both groups (Table S6). Therefore, two cytoplasms coexist in the neo-Sha population, and their distribution is strongly correlated with nuclear haplotypes.

## Discussion

Here, we report a CMS system revealed by combining the cytoplasmic and nuclear backgrounds of two distantly related accessions of *Arabidopsis thaliana*, Sha and Mr-0. The phenotype of the Sha x Mr-0 F1 results from the interaction between naturally evolved loci as demonstrated by the occurrence of other natural accessions possessing a sterility-inducing cytoplasm, or carrying maintainer alleles at genomic positions similar to those of Mr-0.

We compared Sha and Kz-9 cytoplasms to identify the sterilizing factor, taking advantage of their close genetic similarity and difference in sterilizing ability. The genetic variation causing male sterility was likely to fall into either of two types. It could be a variant of a conserved cytoplasmic gene that encodes a dysfunctional product that, in the presence of nuclear maintainer alleles, ultimately leads to pollen abortion. In the G-CMS of sea beet (*Beta vulgaris* ssp. *maritima*), an SNP in exon 2 of the *cox2* gene has been shown to result in the production of a truncated COX2 subunit, and impaired complex IV activity is thought to cause sterility [14]. We analyzed the available results of the Sha sequence [28] and verified, in Sha and Kz-9, the predicted polymorphisms in the exons of mitochondrial and chloroplast genes. Most verified polymorphic positions were identical in both accessions, further demonstrating the close relatedness of their cytoplasms. The only Sha-specific variation found in a coding sequence was synonymous and therefore very unlikely to cause male sterility (Table S4).

Alternatively, the sterilizing factor could be a non-conserved specific ORF likely to result from a mitochondrial genomic rearrangement, as is the case in most of the CMS systems described thus far [18]. With a limited number of mitochondrial markers, we initially detected a structural variation between the Kz-9 and Sha genomes, in the region of *orf240a*. To obtain an overall view of the mitochondrial structural variations between these two accessions, we performed DNA hybridization with 23 mitochondrial probes, which allowed the survey of over three-fourths of the mitochondrial genome. This approach detected a single structural variation between the Kz-9 and Sha mitochondrial genomes. The most relevant difference between the two sequences was the presence of a new ORF in Sha, which we named *orf117Sha*, and whose predicted product is very similar to that of the *orf108* gene previously described in male-sterile *Brassica juncea* carrying the *Moricandia arvensis* cytoplasm [31]. This strongly suggests that *orf117Sha* is the sterilizing factor of the Sha cytoplasm. Homology between sterility genes of different CMS systems is not common, but has been reported previously in two cases. Rapeseed *orf222* and *orf224*, causing *nap* and *pol* CMS respectively, are closely related [33]; the 49 carboxy-terminus residues of Sorghum *orf107* and BoroII rice *orf79* products are 57% identical and 80% similar [34]. Recently, variants of the *orf108* gene were found in the mitochondrial genomes of other Brassicaceae species whose cytoplasms had the same sterilizing effect on *B. juncea* as that of *M. arvensis* [35]. In addition, this study reported that the production of a mitochondria-addressed ORF108 in anthers of transgenic *A. thaliana* plants induces pollen death [35]. Our PCR-based search for *orf117Sha* in the accessions crossed with Mr-0 showed that this mitochondrial ORF is strictly associated with sterilizing cytoplasms. We conclude that *orf117Sha* is most likely the sterilizing factor of these cytoplasms.

We therefore assume that the male-sterile phenotype of Sha x Mr-0 F1 plants results from cryptic CMS. Cryptic CMS has been reported in *M. guttatus* [11] and suspected in *Arabidopsis lyrata* [13]. Nonetheless, although not gynodioecious, these species are mainly or strictly outcrossing. The spread and evolution of a CMS system is generally considered unlikely in a selfing species such as *A. thaliana*, because the low pollen availability from hermaphrodites would limit seed production in female plants. Even though outcrossing has been observed at variable levels in natural populations of *A. thaliana*, its rate rarely exceeds a few percent [36], [37]. However, close relatives of *A. thaliana*, *A. halleri* and *A. lyrata*, are obligate outcrossers due to self-incompatibility, a genetic system that prevents the germination and/or growth of self-pollen [38]. *A. thaliana* lost its outcrossing mating system through the inactivation of genes from the self-incompatibility locus and this loss is thought to have occurred independently in several lineages [39]–[41]. The transition to selfing reproduction has been dated to as recently as 320,000 years ago or at least 1 million years ago [42]–[44]. In any case, these estimations indicate that *A. thaliana* enjoyed a relatively long period of allogamy since its divergence from its self-incompatible sister species – probably over 10 million years ago [45]– until its relatively recent switch to autogamy [46]. It is therefore possible that the CMS system described here controlled sexual polymorphism in *A. thaliana* populations when the species was mainly outcrossing, i.e. before the loss of self-incompatibility. Therefore, the Sha/Mr CMS system provides a valuable tool for exploring the history and evolutionary fate of a CMS system that has become cryptic, and its possible contribution to cytonuclear coevolution.

To gain insight into this history, we analyzed the distribution of the sterility gene in a panel of natural accessions. We found *orf117Sha* in a total of 32 accessions out of the 240 tested (Table S5), a frequency of ∼13% in our sample. It was found mainly associated with the I chlorotype except in two cases, where it was associated with the J chlorotype. The J chlorotype is the closest chlorotype to the root of the phylogeny previously established for *A. thaliana* cytoplasms and therefore considered the most ancient one [27](Figure 6). It is possible that *orf117Sha* appeared in a J-type cytoplasm that subsequently differentiated into an I-type. In this case, I-type cytoplasms devoid of *orf117Sha* (7 cases, including Kz-9, out of 37) would have secondarily lost the sterility gene. It will be interesting to further examine cytoplasmic markers in I-type cytoplasms and to infer their phylogenetic relationships to determine whether the gene was independently lost in this cytoplasmic lineage.

The distribution of *orf117Sha* was not homogeneous among our worldwide sample. It was generally restricted to accessions originating from Central Asia and found exclusively in one nuclear diversity group [24] (Table 4, Figures 5 and 6). In this group, over one-third (35%) of the accessions tested (n = 91) possessed the mitochondrial sterility factor, which strongly suggests that the sterilizing cytoplasm of the Sha/Mr CMS system spread among and was retained in a significant number of populations. In contrast, none of the tested accessions in any of the three other nuclear groups (n = 149) carried this gene (Table 4).

Furthermore, all accessions from our worldwide sample that carry an I-type cytoplasm (n = 37), and most of those carrying a J-type cytoplasm (19 out of 21), regardless of whether they carry *orf117Sha* or not, belong to nuclear diversity group 1 (Table S5, Figure 6). Our previous study did not find any relationships between patterns of cytoplasmic and nuclear diversity [27], but only 95 accessions were analyzed instead of 240 in the present study, and it was based on the analysis of 189 SNPs on 10 nuclear loci, whereas that of Simon *et al.* used 341 SNPs distributed over the entire genome [24].

In addition, we observed a strong correlation between cytotype and nuclear haplotype at a local scale in the neo-Sha natural population. As neo-3 and neo-6 nuclear genotypes are very closely related (15 unshared alleles out of 341 SNPs) [24], it is very unlikely that this correlation results from the recent migration of an unrelated genotype in the neo-Sha population. As far as we can tell from our mitochondrial and chloroplast markers, the two cytoplasmic haplotypes present in the neo-Sha population – I and U– are shared with other accessions, which makes differentiation of the two cytotypes within the population very unlikely. Therefore, we believe that the two cytoplasms have coexisted for a long period in the neo-Sha population. This underlines the relevance of analyzing cytoplasmic polymorphisms in studies describing the genetic structure of *A. thaliana* natural populations.

Sha has been used extensively in crosses with other natural accessions in different laboratories – including ours – involving some 40 different partners ([25], Versailles Arabidopsis Stock Centre unpublished results, http://www-ijpb.versailles.inra.fr/en/cra/cra_accueil.htm ). To our knowledge, no male-sterile F1 have appeared in previous studies. This suggests that nuclear restorers are frequent among *A. thaliana* accessions. We found that Mr-0 and two additional accessions, genetically and geographically closely related to Mr-0, carry maintainers that are dominant on Sha alleles. Recessive restorer alleles are very uncommon; the only reported example that we know concerns alleles of the homozygous lethal *rfl1* restorer for maize CMS-S [47]. Therefore, we do not exclude the possibility that some of the accessions crossed with Sha carry maintainer alleles that are recessive to Sha restorer alleles. Checking for the presence of recessive maintainers requires the observation of F2 or backcrossed progenies. By producing backcross progenies with the seven other accessions of the smallest core-collection of Versailles Arabidopsis Stock Centre [23], we found that Cvi-0 carries recessive maintainers, whereas all six other accessions, namely Blh-1, Bur-0, Ct-1, Ita-0, Jea, and Oy-0 restore fertility in the Sha cytoplasm-induced CMS. It is noteworthy that these accessions belong to genetic diversity groups 2, 3 and 4, and that Ita-0 and Cvi-0 are closely related to Mr-0, according to a previously reported genetic diversity analysis [24]. It remains to be established whether the same maintainer loci are shared between Mr-0 and Cvi-0 and to understand the dominance relationships between these maintainers and Sha restorer alleles. Our results nevertheless indicate that restorer alleles are present across a wide diversity of genotypes, although the sterilizing cytoplasm is restricted to a single nuclear diversity group (Figure 6).

In the reported cases of Mimulus and Helianthus cryptic CMS, the sterilizing cytoplasms are rare and mostly restricted to unique populations, whereas nuclear restorers are common [12], [48]. Several explanations are evoked for this observation: ecological restriction of the spread of the sterilizing cytoplasm, but not for that of Rf genes [12]; or the Rf genes predate the sterilizing cytoplasm [48]. In the case of the Ogura CMS system in wild radish, the cytoplasm had lost its sterilizing capacity, probably after the spread of Rf genes, resulting in hermaphroditic European populations with frequent restorers but devoid of sterilizing cytoplasm [49]. The identification of restorer/maintainer genes, currently underway in our laboratory, and the analysis of their allele distributions are required to address the issue for the Sha/Mr CMS in *A. thaliana*. This knowledge will also allow investigating whether present-day maintainers are ancestral alleles or derived from restorers.

Taken together, our results strongly support the view that *orf117Sha* is the sterilizing factor of an ancient CMS that once drove gynodioecy in *A. thaliana* populations. They raise intriguing questions regarding the evolutionary history of the Sha/Mr CMS system in *A. thaliana*. For example, did the sterilizing cytoplasm continue to spread after it became cryptic? Were gynodioecious populations restricted to the lineage that gave rise to present-day accessions belonging to nuclear diversity group 1? If so, how did restorers invade nuclear lineages that did not coevolve with the sterility-inducing cytoplasm? Alternatively, did the sterility-inducing cytoplasm spread among the common ancestors of present-day accessions and select for restorers before the divergence of different lineages? If so, nuclear diversity groups 2, 3 and 4 might have lost the sterilizing cytoplasm, whereas it was retained in group 1.

In addition, our analyses at two different scales of diversity, at the species level and within a natural population, revealed a preferential association of the sterility-inducing cytoplasm with a particular nuclear diversity group or haplotype. Our present results therefore raise interesting questions on the respective roles of demographic events and selective forces in the coevolution of cytoplasmic and nuclear genomes and shows that the model *A. thaliana* deserves to be studied in this respect.

## Materials and Methods

### Plant Material

*Arabidopsis thaliana* accessions used in this study were obtained from the Versailles Arabidopsis Stock Centre (http://www-ijpb.versailles.inra.fr/en/cra/cra_accueil.htm).

Monte-Rosso (Mr-0) is misspelled ‘Monte-Tosso’ in most stock centers.

598 accessions were previously genotyped with 341 nuclear SNP polymorphisms, among which our worldwide panel of 240. The clustering analysis of the whole dataset resulted in an optimal cluster number of K = 4, thus defining four nuclear genetic groups, among which group 1 predominantly contained accessions originating from Central Asia and Russia [24].

The complete neo-Sha population was kindly provided by Olivier Loudet. It has been partially described in [32], [50]. Information on this population is available at http://www.inra.fr/vast/collections.htm.

Plants were grown in the greenhouse under standard conditions and watered when necessary with tap water, and once a week with a standard nutrition medium.

Crosses were carried out by emasculating flowers of the female parent before anthesis (prior to flower opening) under dissecting microscope and depositing pollen from opened flowers of the male parent on the stigma. Emasculation of male-sterile plants was not necessary. In crosses involving male-sterile mothers, the pollen of the male parent was put on the stigma of recently opened flowers of the female parent.

### DNA Extractions

Progeny genotyping was performed in 96-well plates on genomic DNA extracted according to the procedure described in [51]. Extractions of individual accessions were carried out according to the procedure described in [52].

### PCR Analyses

Primers used for chlorotyping and for amplification of *orf240a* and *orf107d* are described in [27]. Other primers used in this study are listed in Table S7.

The same PCR primers were used for the detection of *orf117Sha* and the production of the *orf117Sha* probe.

### DNA Hybridizations

Total genomic DNAs, or DNAs extracted from enriched mitochondria pellets obtained by differential centrifugation as described in [53], were digested overnight with restriction enzymes in the buffer recommended by the manufacturer (Fermentas) with 0.17 mg.L^-1^ RNase A and 2 mM spermidine. After electrophoresis in 0.8% agarose, digested DNAs were blotted on Genescreen hybridization transfer membranes and hybridized. Probes were labeled with α^ 32^P dCTP by random-priming synthesis with Prime-a-Gene Labeling system (Promega) and purified on Micro Bio-Spin P-30 columns (Bio-Rad) to eliminate unincorporated nucleotides. Either a single probe or a mix of five different probes was used. In the latter case, the probes were individually labeled and purified and mixed prior to hybridization. Hybridizing fragments were revealed by X-ray film exposure.

### Organelle SNP Analysis

We recovered predicted SNPs with a quality score of 20 or more for the Sha chloroplast and mitochondrial genomes on the 1001 genome database (http://1001genomes.org/data/MPI/MPICao2010/releases/current/strains/Sha/). As some polymorphic positions appeared ambiguous, we also recovered the raw data of the Sha Illumina sequencing [28] and aligned them onto the reference sequences for *A. thaliana* chloroplast (NC_000932) and mitochondrial (NC_001284) genomes using BWA [54]. Alignments were visualized using IGV (Integrative Genomics Viewer) software (http://www.broadinstitute.org/igv) and the polymorphic positions were checked manually. This survey also detected predicted polymorphisms that had not been previously predicted, such as small (1–4 bp) insertions. Whenever a predicted polymorphism was located in an exon, we developed PCR primers to check it by sequencing (Table S7). The snpEff tool, v. 2.0.4 (http://snpeff.sourceforge.net/) analyses the input variants and give the prediction of their effect on genes. When several verified polymorphisms affected the same codon, their joint effect was edited manually.

### Sequencing of the Sha and Kz-9 mt Rearranged Regions

Approximately 500 ng of total genomic DNA of Sha was digested with *Bam*HI, *Eco*RI, *Hin*dIII or *Spe*I and subsequently extracted with phenol:chloroform:isoamyl alcohol (25∶24:1) and precipitated with ethanol. After centrifugation, pellets of digested DNA were resuspended in 100 µL of water. Half of the solution underwent ligation with 3 units of T4 DNA ligase in a total volume of 200 µL of the buffer recommended by the manufaturer (Fermentas), at 22°C for 6 hours. The other half was kept for ligase-free controls. Five µL, 1 µL, 0.5 µL and 0.1 µL of the ligations, and equivalent amounts of ligase-free controls, were used in PCR reactions with appropriate divergent primers according to the target region and the restriction enzyme (Table S7). Large amplification products (>3 kb) were obtained according to the procedure described in [55]. Amplification products observed only with ligated substrates at the approximately expected size (according to DNA hybridization results) were sequenced using PCR primers.

Once the origin of the different regions of rearranged sequences had been identified, direct PCR amplification followed by sequencing with PCR primers was used to fill gaps.

### Chlorotyping

Chlorotypes were determined by first sequencing the ndhC-trnV intergenic region [27]. The ndhC-trnV haplotype distinguishes among previously described A, D, H, I, J, L, P, Q, V, X, AA, AH, AC, AD, AE, AJ, AK, AL, and AN chlorotypes. Accessions with any of these haplotypes were thus considered possessing a chlorotype similar to the previously described one (designated A-like, D-like, etc.). Other ndhC-trnV haplotypes corresponded to chlorotype groups (B-E-F-G; K-N-O; R-S-T-U; W-Y-Z; AF-AK-AM; AG-AI) and some were further distinguished by sequencing one or two other chloroplast intergenic regions. For example, B, E, F and G chlorotypes were distinguished by sequencing the NdhF-rpl32 intergenic region.

## Supporting Information

Figure S1 Phenotypes of reciprocal F1s from Sha and Etna-2 parents.
(TIFF)

Figure S2 Genotyping of the (Sha x Etna-2) x Sha backcross population. Legend as for Figure 2. See also Table S2.
(TIFF)

Figure S3 Seedling phenotypes segregating in the progeny of Sha x (Mr-0 x Etna-1). Seeds from the cross were sown directly on soil in the greenhouse. The seedlings showing the typical Sha x Etna-1 phenotype are indicated by red arrows. A few days after the shown stage, they bleached and finally died. Their normal siblings have a wild-type phenotype.
(TIFF)

Figure S4 Phenotypes observed in the Sha x (Mr-0xEtna-1) progeny. Typical sterile (A) and partially fertile (B) plants of the Sha x (Mr-0xEtna-1) progeny.
(TIF)

Figure S5 Comparison of DNA hybridizations between Sha and Kz-9 with *trnK* (A) and *orf122c* (B) probes. Total genomic DNA from Sha and Kz-9 underwent restriction enzyme digestion, 0.8% agarose gel electrophoresis, and were blotted onto a membrane. ^32^P-labeled, PCR-amplified gene fragments were used as probes. Red stars highlight RFLPs.
(TIFF)

Figure S6 Comparison of DNA hybridizations between Sha and Kz-9 with *cob* (A) and *orf117Sha* (B) probes. Total genomic DNA from Sha and Kz-9 underwent restriction enzyme digestion, 0.8% agarose gel electrophoresis, and were blotted onto a membrane. ^32^P-labeled, PCR-amplified gene fragments were used as probes. Red stars highlight RFLPs.
(TIFF)

Figure S7 Comparison of DNA hybridizations between Sha and Kz-9 with mixed probes *nad3*, *atp6-1*, *atp8, nad7, cox1* (A) and mixed probes *rpl5, cox3, atp9, ccb203, rps7* (B). Mt-enriched genomic DNA from Sha and Kz-9 underwent restriction enzyme digestion, 0.8% agarose gel electrophoresis, and were blotted onto a membrane. ^32^P-labeled, PCR-amplified gene fragments were used as probes. Several probes were combined in the same hybridization experiment. Red stars highlight RFLPs.
(TIFF)

Figure S8 Comparison of DNA hybridizations between Sha and Kz-9 with mixed probes *rpl16, cox2, nad6, nad4, atp1* (A) and mixed probes *orf107a, ccmC, nad4L, rpl2, rps4* (B). Mt-enriched genomic DNA from Sha and Kz-9 underwent restriction enzyme digestion, 0.8% agarose gel electrophoresis, and were blotted onto a membrane. ^32^P-labeled, PCR-amplified gene fragments were used as probes. Several probes were combined in the same hybridization experiment. The membrane used in Figure S7 B was dehybridized and rehybridized with probes shown in B.
(TIFF)

Table S1 Genotypes of the (Sha x Mr-0) x Sha backcross population. For each genotyped marker, the distribution of genotypes and Chi-square statistics for a balanced 1∶1 proportion of genotypes are given for the whole population, and both subpopulations of fertile and sterile plants.
(XLS)

Table S2 Genotypes of the (Sha x Etna-2) x Sha backcross population. For each genotyped marker, the distribution of genotypes and Chi-square statistics for a balanced 1∶1 proportion of genotypes are given for the whole population, and both subpopulations of fertile and sterile plants.
(XLS)

Table S3 Genotyping and phenotyping of the Sha x (Mr-0xEtna-1) progeny. Plants that survived after germination were genotyped for CAPS markers developed from two SNPs (mk10070 & mk30220; https://www.versailles.inra.fr/ijpb/crb/anatool [24]) closely linked to the Mr-0 maintainer loci, and that could distinguished Sha/Etna-1 from Sha/Mr-0 heterozygotes. The fertility of plants was scored as their ability to produce seeds by self-pollination in our standard greenhouse conditions (see also Figure S4).
(XLS)

Table S4 Polymorphisms in mitochondrial (mt) and plastid (pt) genes confirmed by sequencing in Sha and Kz-9.
(XLS)

Table S5 Distribution of *orf117Sha* among worldwide accessions.
(XLS)

Table S6 Distribution of nuclear and cytoplasmic polymorphisms in the neo-Sha natural population. The genotypes at tested msat markers were arbitrarily labeled A (neo-3-like) or B (neo-6-like) (Loudet and Trontin, personal communication).
(XLS)

Table S7 Primers used in PCR amplifications. When necessary, PCR products were sequenced with either or both PCR primers.
(XLS)
