# Supplementary figures and images for: A Cryptic Cytoplasmic Male Sterility Unveils a Possible Gynodioecious Past for Arabidopsis thaliana
Source: PLoS One. 2013 Apr 29;8(4):e62450. doi: 10.1371/journal.pone.0062450 (PMC3639211; doi:10.1371/journal.pone.0062450)

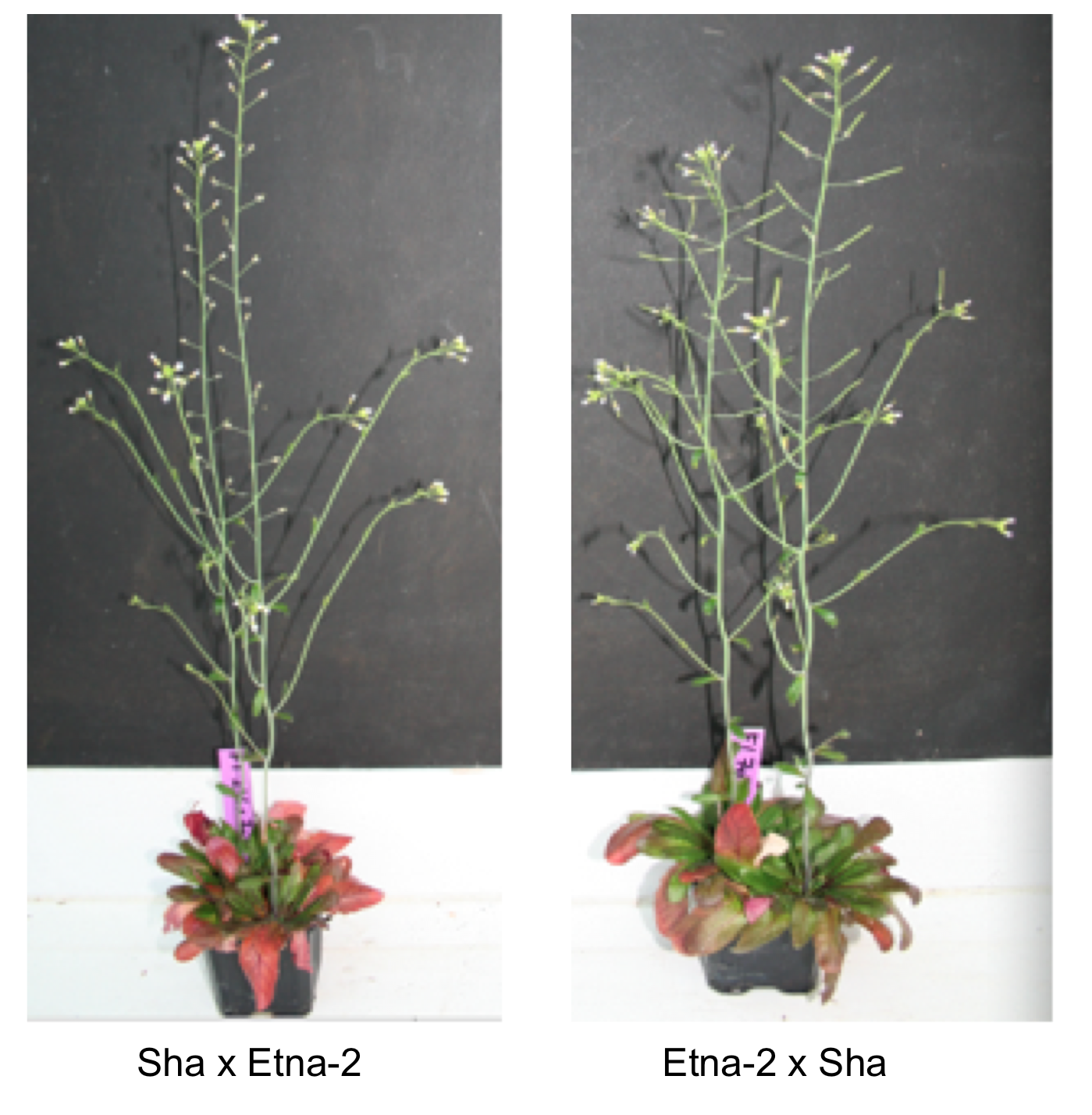

Supplement: Figure S1 — Phenotypes of reciprocal F1s from Sha and Etna-2 parents. (TIFF) [file pone.0062450.s001.tiff]

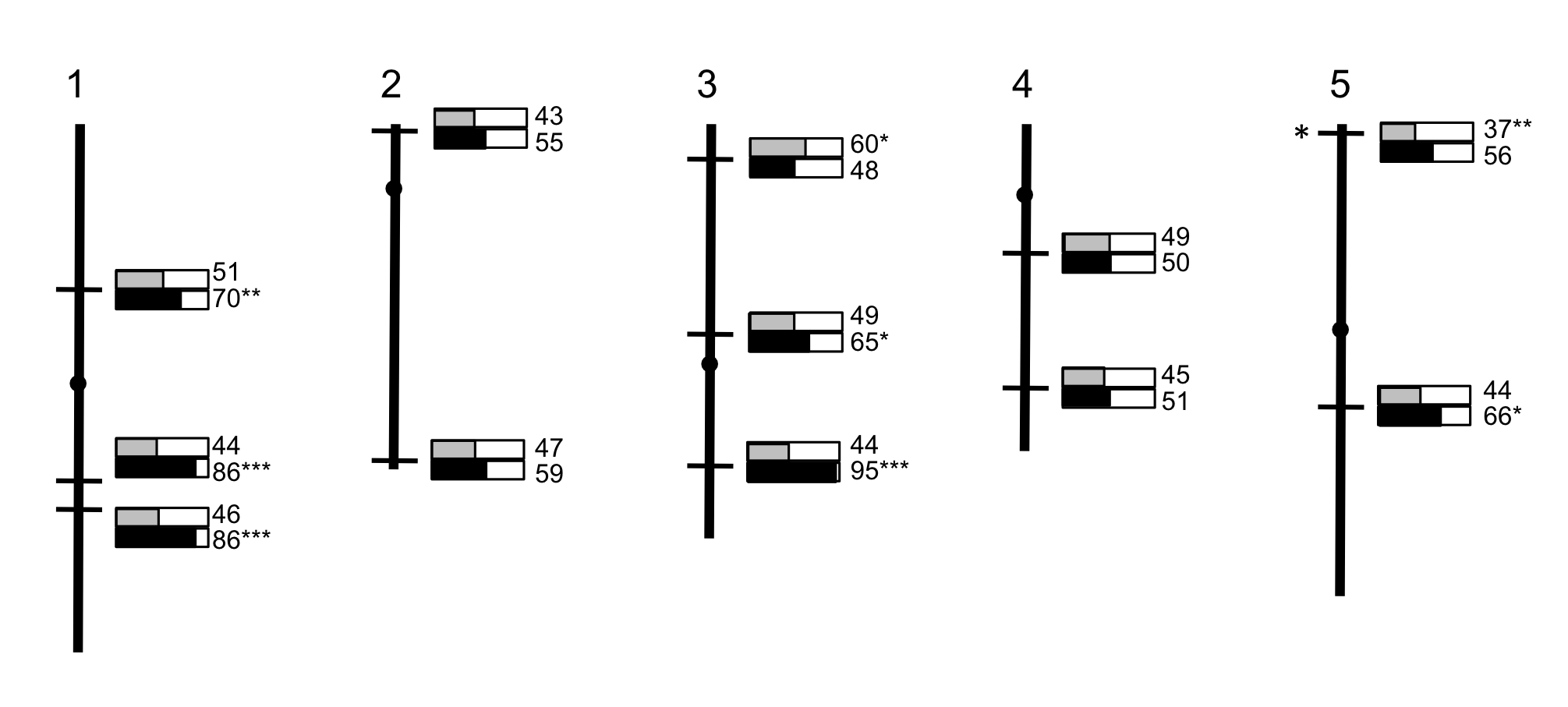

Supplement: Figure S2 — Genotyping of the (Sha x Etna-2) x Sha backcross population. Legend as for Figure 2. See also Table S2. (TIFF) [file pone.0062450.s002.tiff]

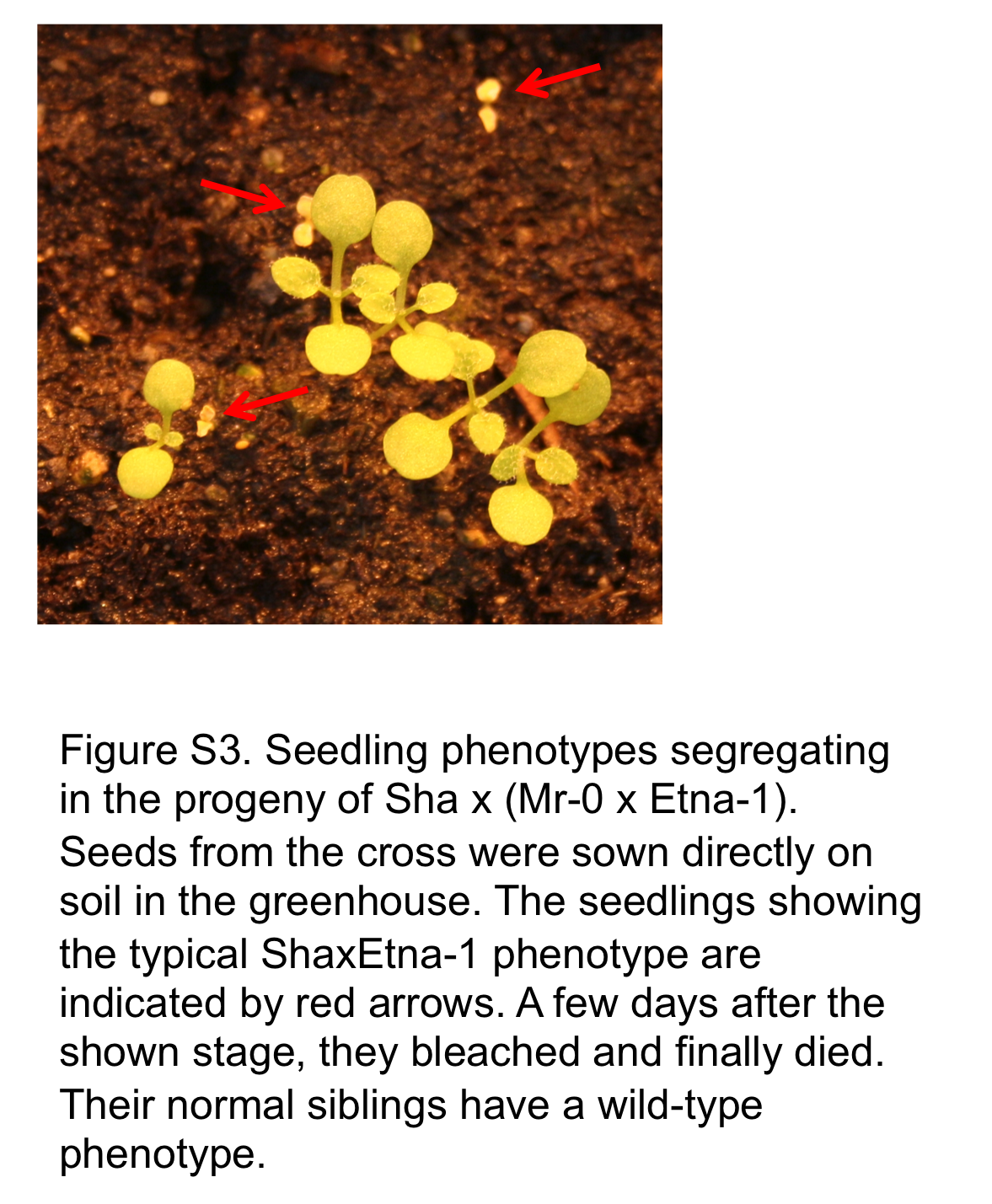

Supplement: Figure S3 — Seedling phenotypes segregating in the progeny of Sha x (Mr-0 x Etna-1). Seeds from the cross were sown directly on soil in the greenhouse. The seedlings showing the typical Sha x Etna-1 phenotype are indicated by red arrows. A few days after the shown stage, they bleached and finally died. Their normal siblings have a wild-type phenotype. (TIFF) [file pone.0062450.s003.tiff]

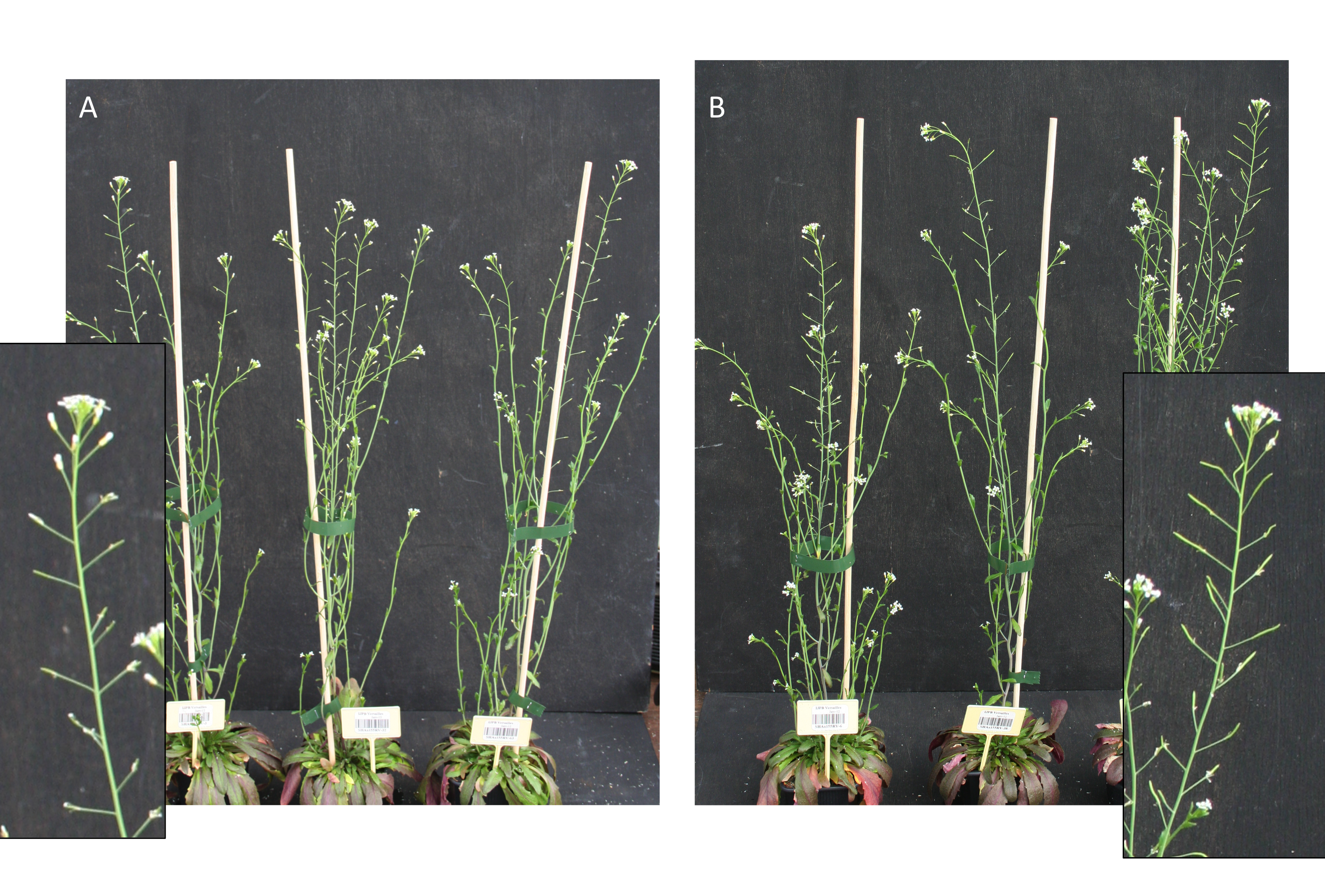

Supplement: Figure S4 — Phenotypes observed in the Sha x (Mr-0xEtna-1) progeny. Typical sterile (A) and partially fertile (B) plants of the Sha x (Mr-0xEtna-1) progeny. (TIF) [file pone.0062450.s004.tiff]

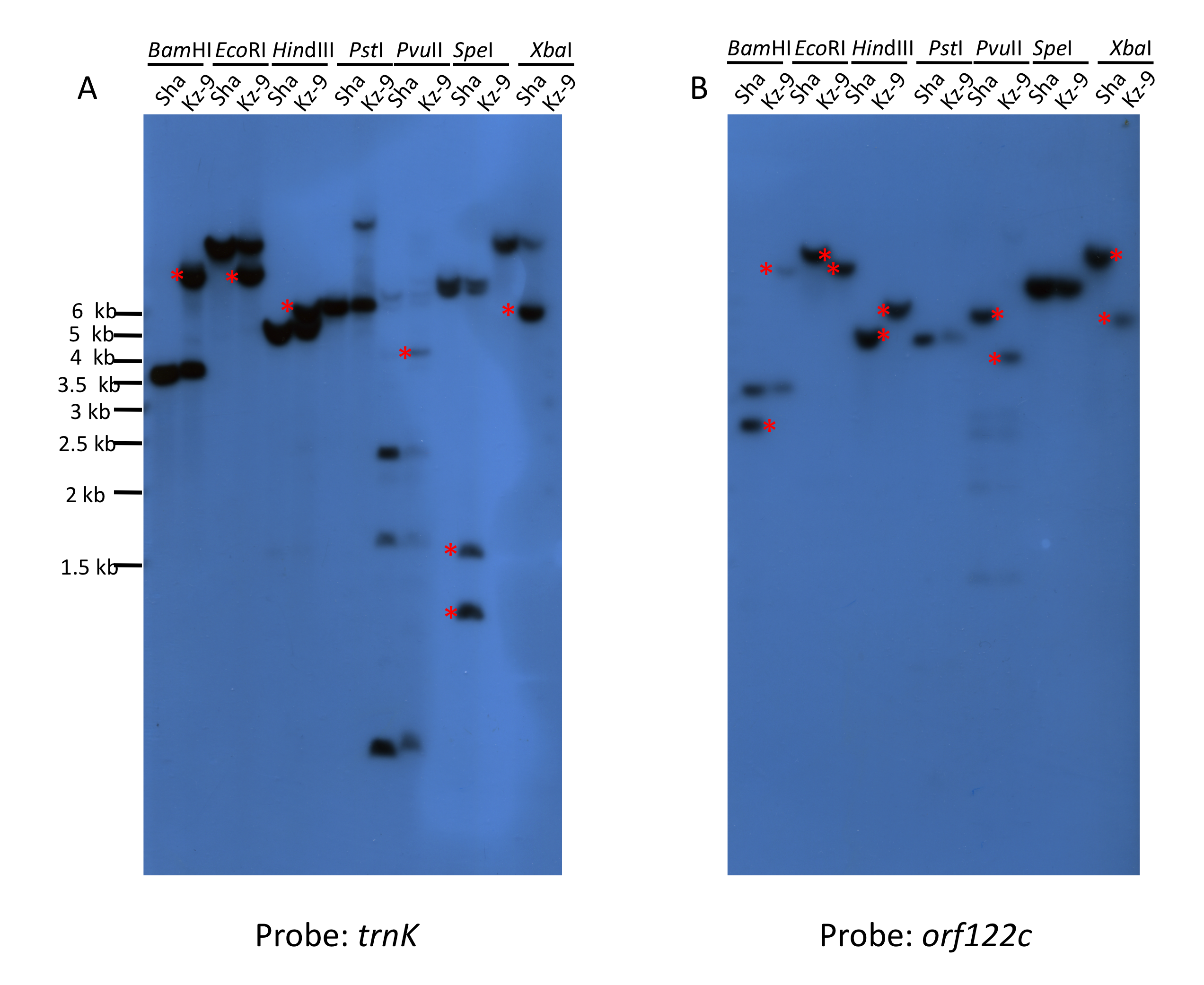

Supplement: Figure S5 — Comparison of DNA hybridizations between Sha and Kz-9 with trnK (A) and orf122c (B) probes. Total genomic DNA from Sha and Kz-9 underwent restriction enzyme digestion, 0.8% agarose gel electrophoresis, and were blotted onto a membrane. 32P-labeled, PCR-amplified gene fragments were used as probes. Red stars highlight RFLPs. (TIFF) [file pone.0062450.s005.tiff]

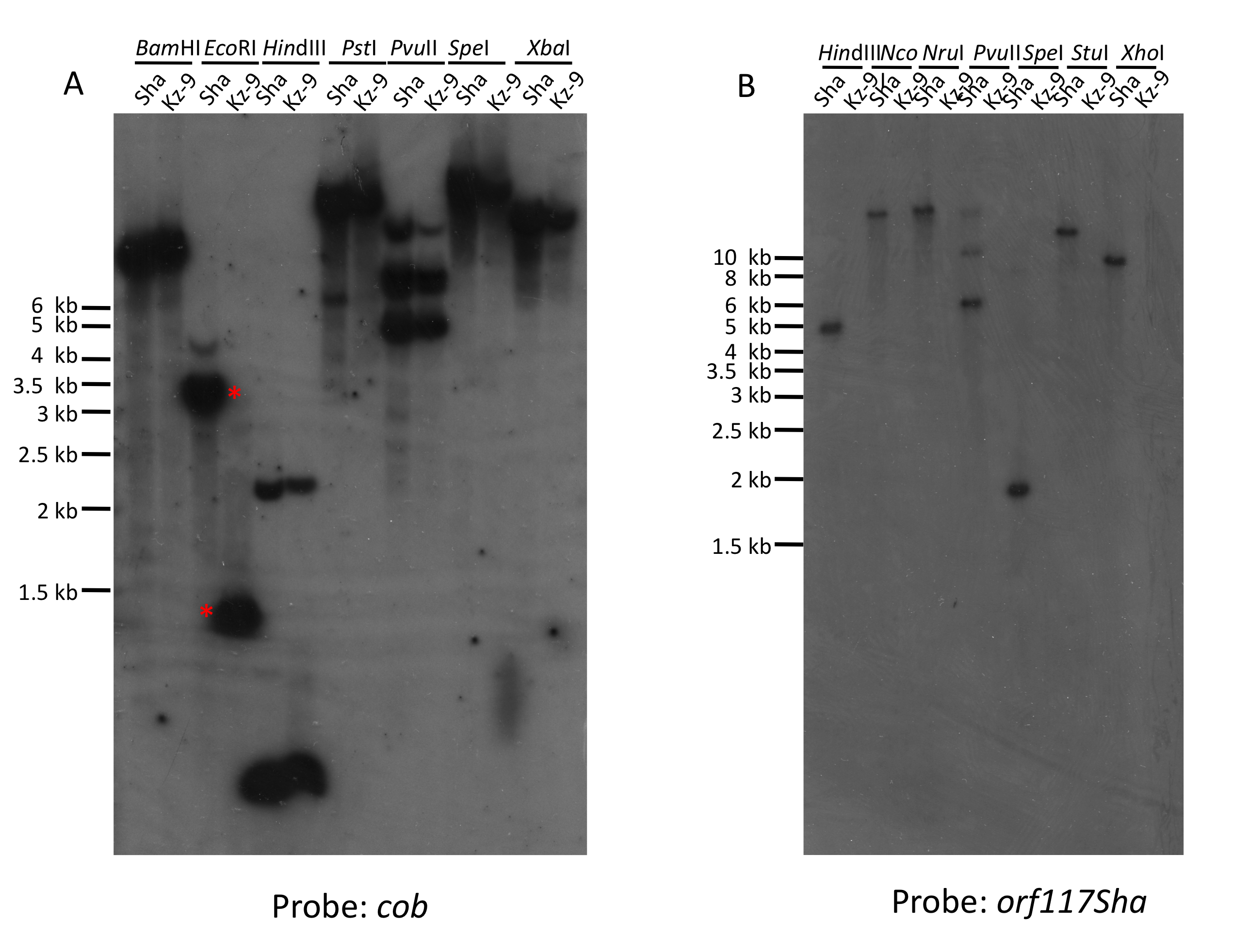

Supplement: Figure S6 — Comparison of DNA hybridizations between Sha and Kz-9 with cob (A) and orf117Sha (B) probes. Total genomic DNA from Sha and Kz-9 underwent restriction enzyme digestion, 0.8% agarose gel electrophoresis, and were blotted onto a membrane. 32P-labeled, PCR-amplified gene fragments were used as probes. Red stars highlight RFLPs. (TIFF) [file pone.0062450.s006.tiff]

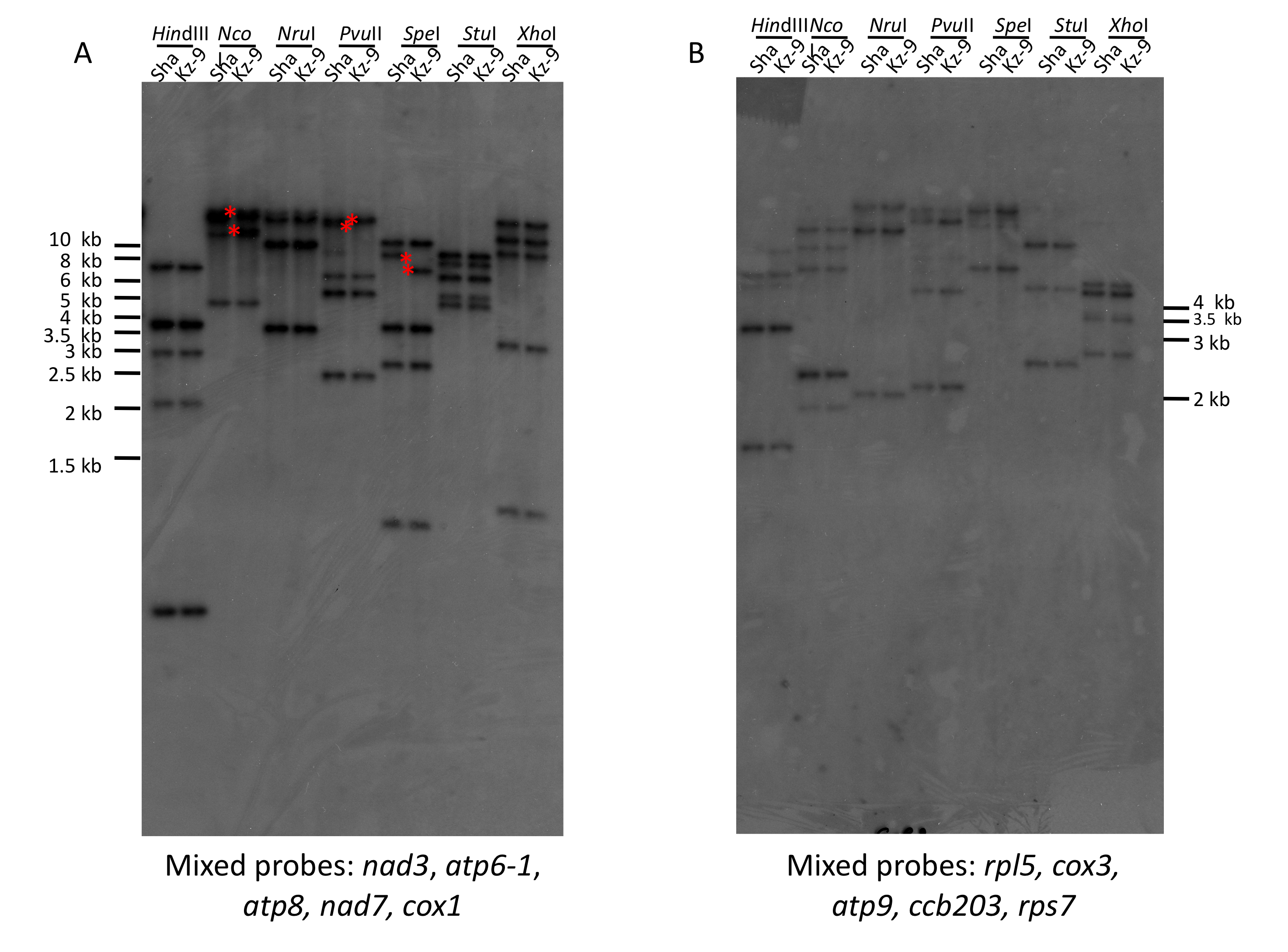

Supplement: Figure S7 — Comparison of DNA hybridizations between Sha and Kz-9 with mixed probes nad3, atp6-1, atp8, nad7, cox1 (A) and mixed probes rpl5, cox3, atp9, ccb203, rps7 (B). Mt-enriched genomic DNA from Sha and Kz-9 underwent restriction enzyme digestion, 0.8% agarose gel electrophoresis, and were blotted onto a membrane. 32P-labeled, PCR-amplified gene fragments were used as probes. Several probes were combined in the same hybridization experiment. Red stars highlight RFLPs. (TIFF) [file pone.0062450.s007.tiff]

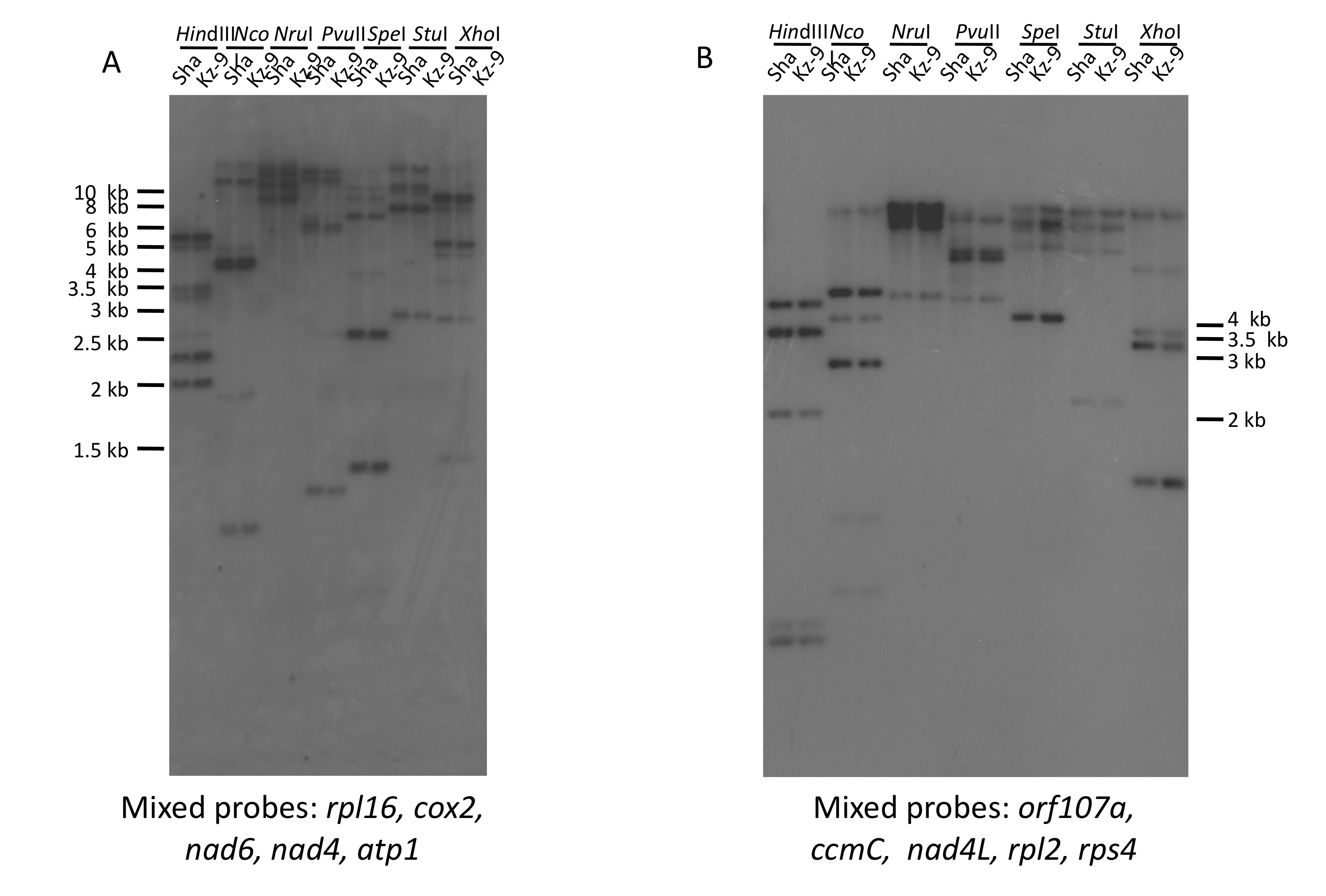

Supplement: Figure S8 — Comparison of DNA hybridizations between Sha and Kz-9 with mixed probes rpl16, cox2, nad6, nad4, atp1 (A) and mixed probes orf107a, ccmC, nad4L, rpl2, rps4 (B). Mt-enriched genomic DNA from Sha and Kz-9 underwent restriction enzyme digestion, 0.8% agarose gel electrophoresis, and were blotted onto a membrane. 32P-labeled, PCR-amplified gene fragments were used as probes. Several probes were combined in the same hybridization experiment. The membrane used in Figure S7 B was dehybridized and rehybridized with probes shown in B. (TIFF) [file pone.0062450.s008.tiff]
